# Quantitative Mass Spectrometry-Based Proteomics for Biomarker Development in Ovarian Cancer

**DOI:** 10.3390/molecules26092674

**Published:** 2021-05-03

**Authors:** Joohyun Ryu, Stefani N. Thomas

**Affiliations:** Department of Laboratory Medicine and Pathology, University of Minnesota School of Medicine, 420 Delaware St SE, MMC 609, Minneapolis, MN 55455, USA; jryu@umn.edu

**Keywords:** ovarian cancer, biomarker, proteomics, mass spectrometry

## Abstract

Ovarian cancer is the most lethal gynecologic malignancy among women. Approximately 70–80% of patients with advanced ovarian cancer experience relapse within five years and develop platinum-resistance. The short life expectancy of patients with platinum-resistant or platinum-refractory disease underscores the need to develop new and more effective treatment strategies. Early detection is a critical step in mitigating the risk of disease progression from early to an advanced stage disease, and protein biomarkers have an integral role in this process. The best biological diagnostic tool for ovarian cancer will likely be a combination of biomarkers. Targeted proteomics methods, including mass spectrometry-based approaches, have emerged as robust methods that can address the chasm between initial biomarker discovery and the successful verification and validation of these biomarkers enabling their clinical translation due to the robust sensitivity, specificity, and reproducibility of these versatile methods. In this review, we provide background information on the fundamental principles of biomarkers and the need for improved treatment strategies in ovarian cancer. We also provide insight into the ways in which mass spectrometry-based targeted proteomics approaches can provide greatly needed solutions to many of the challenges related to ovarian cancer biomarker development.

## 1. Overview of Ovarian Cancer

Ovarian cancer is a relatively rare cancer compared to other cancers, accounting for approximately 3% of cancers in women; however, it is the fifth most common cause of cancer death in women [1]. According to the National Cancer Institute’s Surveillance, Epidemiology, and End Results (SEER) Program, there were an estimated 21,750 new cases and 13,940 ovarian cancer-related deaths in the United States in 2020, representing 1.2% of all new cancer cases and 2.3% of all cancer deaths. Globally, there are approximately 300,000 new cases and roughly 180,000 deaths from ovarian cancer each year. Ovarian cancer is the most lethal gynecologic malignancy cancer among women of advanced age (>40) in developed countries [2,3]. The mortality rate of ovarian cancer is three times higher than that of breast cancer even though the occurrence of ovarian cancer is lower than that of breast cancer [4,5].

Ovarian cancer is staged using the International Federation of Gynecology and Obstetrics (FIGO) system, which is based on surgical results considering the extent of the primary tumor, the presence of cancer in the lymph node, and metastasis to other parts of the body [6,7]. Ovarian cancer has four stages: stage I (early stage) to IV (advanced stage). In stage I, the cancer is limited to the ovaries and has not spread to other regions such as the abdomen, pelvis, or lymph nodes. In stage II, growth of the cancer involves one or both ovaries and fallopian tubes with pelvic extension. In stage III, growth of the cancer involves one or both ovaries or fallopian tubes and the cancer has either spread to the lining of the abdomen or to the lymph nodes in the abdomen. Stage IV is the most advanced stage of ovarian cancer, in which the cancer has metastasized to distant areas or organs in the body [8].

Ovarian cancer treatment plans and prognosis vary according to the stage of ovarian cancer. Generally, early detection leads to better outcomes. If the disease is diagnosed and treated in stage I, the 5-year survival rate is 93% [9]. However, the lack of a proper screening method and the asymptomatic growth of the cancer are the main reasons why ovarian cancer is often not diagnosed until it has reached an advanced stage. More than 75% of ovarian cancer is diagnosed in stage III or IV. Therefore, ovarian cancer is known as a “silent killer” [5,10,11].

Ovarian cancer is a heterogeneous disease. It encompasses a variety of tumors with various histopathological features and biological behavior [12,13,14]. Ovarian cancer is classified into more than 30 different types, and most of the types are identified according to the type of cells in which the cancer originated. Malignant ovarian tumors originate most commonly from three cell types: epithelial (most common, accounting for 90% of all ovarian cancers), germ, and stromal. Each type of cell develops into a different type of cancer: epithelial ovarian carcinoma (EOC), germ cell tumor, and stromal tumor. EOC, which is the most dangerous of all types of ovarian cancers, develops from the cells covering the outer surface of the ovaries and is classified into five subtypes based on tumor cell histology: high-grade serous, low-grade serous, clear cell, endometrioid, and mucinous ovarian cancers [15,16].

EOC is divided into two groups based on distinct biological features and clinical behaviors: type I and type II tumors [13,17]. Low-grade serous ovarian carcinoma (LGSOC), clear cell, low-grade endometrioid, and mucinous carcinomas are classified as type I tumors, which are associated with continuous ovulation, inflammation, and endometriosis [17,18,19]. Type I tumors grow slowly, cause fewer clinical symptoms, and are frequently diagnosed at an early stage, representing low-grade disease [14,17]. Therefore, type I tumors present with an excellent prognosis. However, type I tumors are often resistant to standard chemotherapy [20]. Type I tumors are also characterized by somatic mutations in *KRAS*, *BRAF*, *PTEN*, *PIK3CA, CTNNB1*, *ARID1A*, and *PPP2R1A* genes [21,22,23]. Mutations in *p53* and *BRCA* genes are rare in type I tumors [17]. Type II EOC tumors are comprised of high-grade serous ovarian carcinoma (HGSOC), high-grade endometrioid carcinoma, undifferentiated carcinoma, and carcinosarcoma. Type II tumors grow fast, are highly aggressive, and present in an advanced stage, representing low survival [17,19]. HGSOC, which is the most common subtype among type II tumors, shows relative chromosomal instability compared to type I tumors and is associated with mutations in *p53* (96%) and *BRCA* genes (22%) [13,24,25].

First-line treatment for ovarian cancer includes cytoreductive surgery followed by chemotherapy combining a platinum compound (cisplatin or carboplatin) and taxane (paclitaxel or docetaxel) [1]. Despite significant advances in first-line treatment in the past few years, the survival rates have improved only slightly. This is due to late diagnosis and a lack of effective second-line treatment for patients with advanced ovarian cancer. Most patients with advanced ovarian cancer such as HGSOC seem to respond well to first-line treatment, but the effects are not typically long-lasting with many of these patients requiring further treatment [3,16]. Approximately 70–80% of patients with advanced ovarian cancer experience relapse within five years and develop platinum-resistance [26,27]. The life expectancy of most patients with platinum-resistance or platinum-refractory disease is less than one year [3,28]. Therefore, it is essential to develop new strategies for the treatment of advanced stage ovarian cancer, which is associated with recurrence.

## 2. Targeted Therapies for Ovarian Cancer

Targeted therapies, including poly (ADP-ribose) polymerase (PARP) inhibitors and immunotherapy, have shown promise in improving outcomes in recurrent ovarian cancer [29,30]. The use of PARP inhibitors as first-line therapy and maintenance therapy has improved outcomes for women with newly diagnosed advanced ovarian cancer [31]. PARP inhibitors target DNA repair pathways including base excision repair, homologous recombination repair and non-homologous end-joining. Therefore, mutations of *BRCA* genes or other genes involved in homologous recombination repair deficiency, confer sensitivity to PARP inhibitors [16].

Three PARP inhibitors (Olaparib, Rucaparib, and Niraparib) are approved by the U.S. Food and Drug Administration (FDA) as treatment or maintenance therapy for patients with HGSOC who undergo recurrence following platinum-based chemotherapy and harbor germline and/or somatic mutations of *BRCA* genes [30,32]. Although *BRCA1*- or *BRCA2*-deficient cancer cells exhibiting defects in homologous recombination (HR deficiency) are hypersensitive to PARP inhibitors [33,34] through the mechanism of synthetic lethality [35], acquired resistance to PARP inhibitors was observed in patients with HR deficient HGSOC. Given the function of *BRCA1* or *BRCA2* in HR repair [36] and maintaining replication fork stability [37], multiple mechanisms are involved in resistance to PARP inhibitors and cancer cell survival. These include: (1) secondary reversion mutations in restoring wild-type *BRCA1* or *BRCA2* function in HR repair either by open reading frame (ORF) restoration or somatic reversion of inherited mutations in *BRCA1*- or *BRCA2*-deficient cancer cells [38,39,40]; (2) restoration of HR repair by epigenetic reversion of *BRCA1* promoter hypermethylation [37]; (3) hypomorphic alleles such as *BRCA1*-C61G mutation [41]; (4) stabilization of mutant *BRCA1* harboring mutations in its C-terminal (BRCT) domain [42]; (5) restoration of HR repair by loss of PARP1 expression itself [37] and DNA repair proteins such as 53BP1 [43,44] or REV7 [45,46]; and (6) other alternative mechanisms regardless of restoration of HR repair such as increased expression of ABC transporters (e.g., P-glycoprotein (PgP) efflux pump) [47], protection of replication fork stability [48], loss of PARG [49], activation of RAS pathway due to RAS proteins (e.g., KRAS mutant) [50], and activation of the PI3K/AKT pathway [51]. Combating PARP inhibitor resistance can be achieved through combination therapies. Therefore, understanding the detailed mechanisms that cause PARP inhibitor resistance and identifying prognostic biomarkers that predict PARP inhibitor resistance will be needed to overcome PARP inhibitor resistance.

Immunotherapy is a new treatment in advanced ovarian cancer such as HGSOC. This treatment modality uses the body’s own immune system to kill cancer cells. In the past few years, immune-checkpoint inhibitors which target immune-checkpoint pathways including cytotoxic T lymphocyte-associated protein 4 (CTLA-4) and PD-1/PD-L1 have been developed in advanced ovarian cancer [16]. Despite encouraging results with immunotherapy in melanoma, non-small cell lung cancer, kidney, and urothelial cancer, immune-check point inhibitors as mono-immunotherapy present modest response rates in ovarian cancer [29,52,53]. Further options for the application of immunotherapy in ovarian cancer include a combination of immune-checkpoint inhibitors with chemotherapy or targeted therapy such as anti-angiogenic agents and PARP inhibitors, as well as a combination of immune-checkpoint inhibitors which target different mechanisms in the immune system. Several clinical trials are currently in progress testing these novel treatment approaches [28].

## 3. Overview of Ovarian Cancer Biomarkers

The National Cancer Institute (NCI) defines a biomarker as a biomolecule found in blood, other body fluid, or tissues which is a sign of a normal or abnormal process, or of a condition or disease, which may be used to determine how well the body responds to a treatment for a condition or disease. A biomarker is effectively an indicator of a normal biological process, pathogenic process, or pharmacological response to a therapeutic intervention [54]. Therefore, cancer biomarkers can clarify the development and progression of cancer, predict prognosis and response to therapy, and monitor the risk of recurrence [55].

There are several types of biomarkers for susceptibility/risk assessment, early detection, diagnosis, prognosis, prediction, and monitoring [56]. The intended purpose or utility of a biomarker should be taken into consideration during the development process. Susceptibility/risk assessment biomarkers identify cancer susceptibility, representing a potential risk of developing cancer. These biomarkers do not provide information regarding whether a person has cancer at the stated point in time. Biomarkers for early detection, which reflect the presence of cancer, are useful in screening patients to discover cancer at an early stage. Generally, patients who have been informed that they have a high risk based on the expression of a susceptibility/risk assessment biomarker are recommended to undergo frequent screening tests for the early detection of cancer. Diagnostic biomarkers help to identify histopathologic characteristics that correlate with the presence or absence of cancer. Prognostic biomarkers are used to analyze the risk of a patient’s clinical outcome in cancer recurrence or cancer progression, regardless of therapy. For example, *BRCA1/2* mutations are prognostic biomarkers; women who harbor mutations of *BRCA* genes are expected to have a high risk of developing breast or ovarian cancer in the future [57]. Predictive biomarkers are used to predict a patient’s response to a specific treatment or monitor the effectiveness of the treatment, thereby guiding treatment decisions. Monitoring biomarkers can reflect a patient’s disease risk or disease status through long-term monitoring of biomarkers. The blood level of CA 125 (Cancer antigen 125), which is among the most widely used ovarian cancer biomarkers, is used to monitor ovarian cancer [58]. More accurate diagnoses as well as more effective and personalized treatment can be achieved by using these types of biomarkers.

In general, cancer is the most common genetic disease that can be attributed to genetic mutations or chromosomal abnormalities. Such genomic instability in cancer cells leads to the alteration of cellular processes, resulting in an uncontrollable state in cancer cell growth [54,59]. As a consequence, scientists have endeavored to identify cancer biomarkers using genomics approaches such as next-generation sequencing technologies [60,61]. These large-scale DNA sequencing efforts have accomplished progress in selecting targeted therapy for personalized treatment approaches based on the genomic profile of cancers [62,63]. However, genomic profile-based targeted therapy has not been as effective as expected, largely due to the development of drug resistance [64,65,66,67]. Genomic alterations are not the only factors that determine the phenotype of cancer cells.

Genes are expressed as multiple proteins with different sequences and activities due to alternatively splicing. Protein expression and function also depend on the transcript levels of their corresponding genes and the translational efficiency. Proteins undergo post-translational modifications (PTMs) to form mature proteins and complexes through protein–protein interaction, which act as important components in cellular processes. Alterations in the expression and activity of proteins can determine the phenotype of the cancer cells as downstream processes of genes [68]. Therefore, quantifying proteomic alterations is as important as identifying genomic changes in the context of targeted therapy.

Proteins have advantages over genes as therapeutic targets in various clinical states: (1) unlike the genome, protein expression is specific to a cell type under specific conditions; (2) environmental influences are more easily reflected in the proteome; (3) the level of protein expression is the result of many upstream processes such as transcription activation, chromatin aberration, transcript degradation and translation efficiency; and (4) proteins are major downstream effectors as well as affecters in various cellular functions [7,68]. Several studies have focused on proteomic alterations during carcinogenesis. The most valuable therapeutic targets to study the phenomenon of chemoresistance in advanced ovarian cancer are proteins: kinase inhibitors, PARP inhibitors, and immune-checkpoint inhibitors.

Typically, biomarker discovery begins with identifying targets that exhibit significant changes in a normal biological process, pathogenic process, or pharmacological response. Each target candidate must be validated before proceeding to clinical studies. This includes the development of sensitive and selective assays in order to monitor specific biomarker candidates.

The process of cancer biomarker development includes biomarker discovery, assay development and analytical validation, clinical validation and utility, and clinical implementation [69]. The first step in biomarker development involves the use of preclinical exploratory methods to identify candidate biomarkers. In this discovery step, a large number of biomarkers can be detected using omics techniques (genomics, epigenomics, transcriptomics, proteomics, and metabolomics) using human or model organism biofluids or tissues. The second step is assay development to detect or measure biomarker candidates, followed by analytical validation. Analytical validation is a process that is employed to demonstrate the sensitivity, selectivity, precision, accuracy, and reproducibility of the assay [69,70]. Typical assay methods that are widely used include antibody-based immunoassays such as western blotting, enzyme-linked immunosorbent assay (ELISA), and immunohistochemistry (IHC). After the analytical validation is established, the biomarker assay must be evaluated to ensure its clinical performance in predicting the clinical outcomes of interest (clinical validation) and improved patient outcomes (clinical utility) [70,71]. Clinical validation is the final step in biomarker development, and it entails the use of an analytically validated assay within a clinical trial. Clinical validation is a process to demonstrate the relevance of the biomarker assay in the clinical condition including disease outcome or treatment outcome. Clinical utility is a measure of whether the clinical use of the biomarker assay improves patient outcome, confirms diagnosis, determines appropriate therapy, or identifies individuals who are at risk of developing disease. The clinical utility of biomarkers is important for conducing risk/benefit assessments when used in individual patient management: administering the right therapy for the right patient [72]. An analytically and clinically validated biomarker assay with acceptable performance characteristics is ready to be implemented in clinical treatment. The four key elements associated with implementing biomarker assay in the clinic are as follows: regulatory approval, commercialization, coverage by health insurance companies, and incorporation in clinical practice guidelines [69]. Strict guidelines have been established for the development of clinically useful biomarkers in precision medicine.

Notable advances in omics technologies have facilitated the discovery of numerous new biomarkers at a rapid pace. However, despite great efforts in cancer biomarker development, only a few biomarkers are currently FDA approved, especially in ovarian cancer [73]. This is due to a gap between initial biomarker discovery in the laboratory and translating the findings into using biomarkers in a clinical setting. There is a lack of validation methods ensuring acceptable performance metrics in sensitivity, selectivity, precision, accuracy, high-multiplexing, and high inter-laboratory reproducibility. Traditional antibody-based immunoassays for biomarker verification and validation have major limitations such as low-multiplexing, low inter-laboratory reproducibility, and lack of specific antibodies, especially for mutated or post-translationally modified peptides. Traditional methods are being increasingly substituted by targeted quantitative proteomics methods as a means of verifying and validating biomarker candidates. Targeted quantitative proteomics will be reviewed in Section 5, “Mass spectrometry for biomarker discovery”. Targeted quantitative proteomics can address many of the challenges related to biomarker development that have created the chasm between biomarker candidate identification and FDA-approval.

## 4. Proteomics in Ovarian Cancer

Single-marker diagnostics are commonly used in the field of ovarian cancer. The serum biomarker CA125 (Cancer Antigen 125 or MUC16) has long been used as the primary ovarian cancer biomarker for preoperative assessment [74,75]. However, CA125 does not have a role in improving ovarian cancer care. The FDA has not approved CA125 for preoperative use, but rather only for cancer surveillance among women with a diagnosis of ovarian cancer [76]. CA125 has a low sensitivity in predicting ovarian cancer at an early stage [75,77,78]. The low sensitivity of CA125 has led to the development of additional serum proteins as candidates for biomarkers that could aid the accuracy of CA125 as an adjunct marker. The combination of CA125 with other biomarkers is an approach that has been pursued in an effort to overcome the limitation of the single use of CA125 [79,80,81].

In vitro Multivariate Index Assays (IVDMIA) include multiple markers to improve the clinical performance of a diagnostic tool. OVA1^®^ was the first IVDMIA comprised of protein biomarkers cleared by the U.S. FDA, and its intended use is to further assess the likelihood of malignancy in women presenting with an ovarian adnexal mass prior to planned surgery [82,83]. OVA1 examines five serum proteins (CA125, transferrin, apolipoprotein A-1, transthyretin, and ß2-microglobulin). OVA1 is not intended to be used to predict the risk of ovarian cancer in asymptomatic patients without pelvic masses. OVA1 is more sensitive than CA125 alone, but it has a lower specificity. This indicates that OVA1 improves detection in women with ovarian cancer prior to surgery but increases false-positive outcomes. OVA1 detects 94% of cancer cases, whereas 77% of cases are detected using CA125 in both pre-menopausal and post-menopausal women [84].

Human epididymis protein 4 (HE4) is a protease inhibitor expressed in malignant epithelial ovarian cells, and it has been identified as the most promising biomarker for ovarian cancer in addition to CA125 [85]. HE4 exhibits the best performance as a single biomarker for distinguishing malignant from benign tumors. In 2008, the FDA approved the use of HE4 in monitoring patients who have a history of ovarian cancer. The detection of HE4 levels in combination with CA125 showed an improvement for the early detection of ovarian cancer [86].

The Risk Of Malignancy Algorithm (ROMA), which measures CA125 and HE4 simultaneously according to a woman’s menopausal status, was approved by the FDA in 2011. The ROMA has a higher sensitivity and specificity for the prediction of ovarian cancer in patients with pelvic masses compared to CA125 alone, allowing it to distinguish the risk of a patient with benign (low risk) and malignant (high risk) conditions, which can improve the early detection of ovarian cancer. In the diagnosis of ovarian cancer, ROMA has a sensitivity of 92.3% with a specificity of 76% in pre-menopausal women and a sensitivity of 100% with a specificity of 74.2% in post-menopausal women [87]. In pre-menopausal women, ROMA values ≥1.31 are highly suggestive of ovarian cancer malignancy, whereas in post-menopausal women, ROMA values ≥2.77 are highly suggestive of ovarian cancer malignancy [18]. However, ROMA is not meant to determine whether a patient requires surgery, and it has not been validated for women previously treated for an ovarian malignancy, women currently being treated with chemotherapy, pregnant women, and women younger than 18 [88,89].

OVA1 has a relatively low specificity (54%). The addition of another biomarker to this panel resulted in the next generation of OVA1, OVERA, which was approved by the FDA in 2016. OVERA includes five biomarkers (CA125, HE4, transferrin, apolipoprotein A-1, and follicle-stimulating hormone). Compared to the biomarker panel that comprises OVA1, transthyretin and ß2-microglobulin are replaced with HE4 and follicle-stimulating hormone. OVERA is intended to distinguish the risk of patients with malignant tumors from those with benign tumors [90]. OVERA has improved specificity (69% vs. 54%) and positive predictive value (40% vs. 31%) compared to OVA1 while maintaining high sensitivity (91%) [91].

The early detection of ovarian cancer is a critical step in mitigating the risk of patients progressing from early stage to advanced stage disease. The best biological diagnostic tool for ovarian cancer seems to be a combination of biomarkers (e.g., IVDMIA). Although the use of multiple biomarkers has less specificity compared to CA125 alone, the benefit of this approach is an increased sensitivity in diagnosing ovarian cancer at an early stage. Thus, when developing methods entailing the use of multiple biomarkers, it is important to improve diagnostic specificity while maintaining high diagnostic sensitivity [92].

Several biomarkers for ovarian cancer have been discovered from various biological sources using methods such as proteomics, lipidomics, and genomics (Table 1). The majority of the protein biomarkers included in this table were identified and characterized using immunoassay-based methods such as enzyme-linked immunoassay (ELISA) and radioimmunoassay (RIA). The National Cancer Institute’s Early Detection Research Network (EDRN) website mentions 616 active clinical trials with 232 biomarkers for ovarian cancer (338 Phase 1 trials, 169 Phase 2 trials, 106 Phase 3 trials, 2 Phase 4 trials, and 1 Phase 5 trial) (https://edrn.nci.nih.gov/data-and-resources/biomarkers (accessed on 28 April 2021). The readers are encouraged to refer to the resource on the EDRN website for additional details regarding the 232 biomarkers included in Table 1. The biological functions of most candidate biomarkers have not been fully elucidated, and most initial diagnoses of ovarian cancer are still dependent on CA125 measurement [93].

## 5. Mass Spectrometry for Biomarker Development

Over the last decade, mass spectrometry-based proteomics has become a promising technology to reveal the quantitative state of the human proteome. This is due in large part to dramatic advances in mass spectrometry instrumentation, peptide/protein identification and quantification algorithms, and bioinformatics computational data analysis. Quantitative proteomics advances in biomedical research provide insights into the dynamic proteome state associated with different biological conditions such as environmental stress, genetic mutations, drug treatment, and diseases [3,94,95,96,97,98,99]. The characterization of the dynamic proteome states can reveal pathogenic mechanisms as well as lead to significant advances in biomarker development by identifying distinct proteins for biomarkers and therapeutic targets.

Mass spectrometry-based proteomics can be categorized into top-down or bottom-up approaches. In top-down proteomics, intact proteins are measured, whereas in bottom-up proteomics, peptides are measured as surrogates for the proteins of interest. Most advanced quantitative proteomics methods for biomarker development are performed using bottom-up proteomics. Two types of quantitative approaches are employed in biomarker studies: untargeted quantitative proteomics for biomarker discovery and targeted quantitative proteomics for biomarker verification and validation. In the workflow for biomarker development, first, a large number of biomarker candidates are identified from a few sample cohorts using untargeted global quantitative proteomics in the discovery stage. Then, a small number of biomarker candidates are further evaluated for reproducibility in a large number of sample cohorts during the verification stage. Finally, the most promising biomarker candidates are validated to assess their sensitivity, specificity, and clinical utility in a much larger number of sample cohorts.

**Figure 1:**
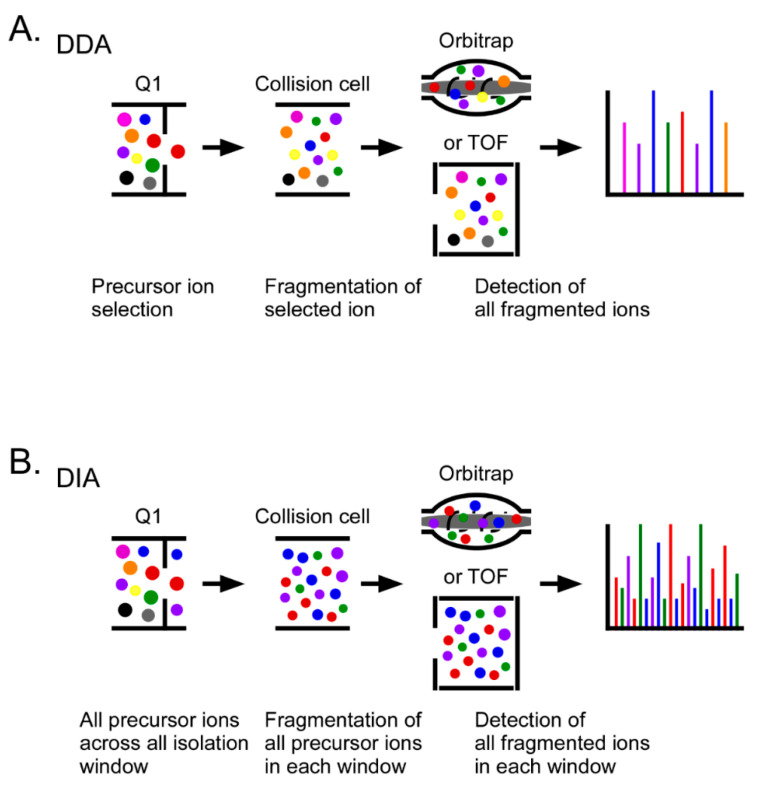
Principles of data-dependent acquisition (DDA) and data-independent acquisition (DIA) in untargeted quantitative proteomics. (**A**) In DDA, precursor ions are stochastically selected on the basis of their signal intensity in Q1 followed by fragmentation of the selected precursor ions in a collision cell. All fragmented ions are separated and detected by a mass analyzer such as an Orbitrap or time-of-flight (TOF) analyzer. (**B**) In DIA, all MS1 precursor ions within pre-defined mass windows are selected in Q1 followed by fragmentation of all precursor ions from each window in a collision cell. The resultant MS2 spectra are comprised of fragment ions from all of the precursor ions in the selected Q1 window.

Untargeted quantitative proteomics approaches that are designed to yield an in-depth unbiased quantitation of the global proteome include label-free and stable isotope labeling techniques using a data dependent acquisition (DDA) mode. Stable isotope labeling uses the mass increase caused by the mass tags with incorporated stable isotopes to quantify peptides at the MS1 full scan (precursor ion) level or peptide fragments at the MS2 (production ion) scan level. There are several strategies to label peptides or proteins with stable isotopes: chemical labeling such as Isobaric Tags for Relative and Absolute Quantitation (iTRAQ) [97,100,101,102] and Tandem Mass Tags (TMT) [103,104,105,106], and metabolic labeling strategies such as Stable Isotope Labeling by Amino acids in Cell culture (SILAC) [107,108,109,110]. Data independent acquisition (DIA) methods can also be used for quantitative studies (Figure 1).

Label-free quantitation uses mass spectrometric signal intensity or peptide spectral counts for peptide and protein quantitation. Label-free quantitation can be performed with DDA and DIA. In DIA, MS2 scans are acquired from all of the detectable peptide ions within the indicated MS1 full scan detection window, as opposed to selecting a fixed number of precursor ions from the most abundant peptides in the MS1 full scan to acquire MS2 scans when using DDA [111]. Quantification is most commonly conducted based on the summation of the intensities of peptide-specific fragment ions in MS2 scans, the identities of which are obtained from ion (spectral) libraries generated using DDA. DIA technologies are mass spectrometer platform-dependent. For example, the representative DIA technologies are BoxCar [112], SWATH [113,114], diaPASEF [115] and MS^E^ and SONAR [116,117,118], which are scan modes for Orbitrap (Thermo, Waltham, MA, USA), TripleToF (Sciex, Framingham, MA, USA), Q-Tof (Bruker, Billerica, MA, USA) and Q-ToF (Waters, Milford, MA, USA) mass spectrometers, respectively.

In biomarker discovery, global proteome analysis using untargeted quantitative proteomics presents promising relative quantitation data for large numbers of biomarker candidates. However, a critical limitation for this approach is the low reproducibility and higher missing values for low abundance peptides/proteins due to the stochastic nature of abundance-based precursor ion selection in the DDA mode, which cannot guarantee that the same peptides will be consistently detected in all analyses. Data imputation or DIA can be used to partially overcome the challenges associated with missing values. This limitation indicates that untargeted global quantitative proteomics approaches are not suitable for the verification and validation of biomarker candidates.

To assess the clinical utility of statistically significant biomarker candidates, it is important to analyze specific peptides/proteins using methods with high sensitivity and reproducibility in a large number of sample cohorts. In this respect, targeted quantitative proteomics, Nature Method’s choice for method of the year in 2012, is a powerful technology for biomarker candidate verification and validation due to its capability to consistently identify and quantify peptides/proteins with higher sensitivity, specificity, reproducibility at a higher sample throughput compared to DDA-based untargeted global quantitative proteomics [119].

**Figure 2:**
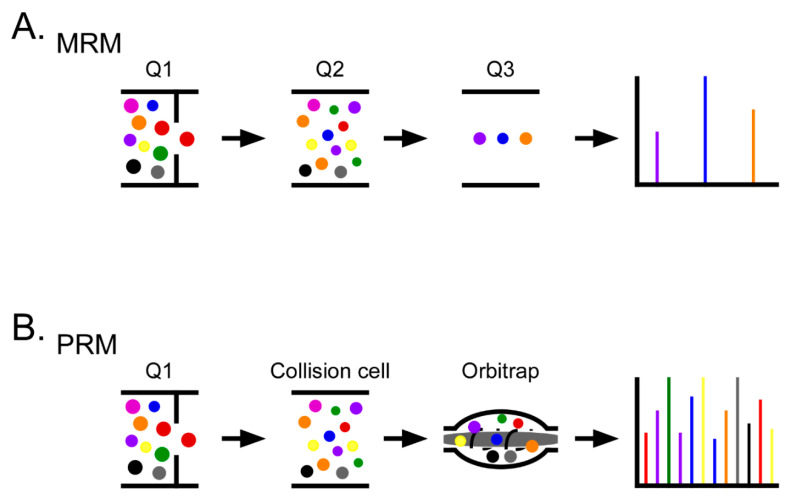
Schematic overview of targeted quantitative proteomics methods. (**A**) In multiple reaction monitoring (MRM), the precursor ion of a pre-defined specific peptide is selected and fragmented in Q1 and Q2, respectively. Pre-defined fragmented ions are selected and detected in Q3. (**B**) Unlike MRM, parallel reaction monitoring (PRM) can detect all fragmented ions generated from precursor ions in parallel using a high resolution accurate mass (HRAM) mass analyzer such as an Orbitrap.

Targeted quantitative proteomics methods include multiple reaction monitoring (MRM) and parallel reaction monitoring (PRM) (Figure 2) [120]. DIA is considered a targeted data extraction quantitative method, as opposed to a targeted data acquisition method. In general, missing values are less of a challenge with DIA methods compared to DDA [119,121]. The relatively lower sensitivity, specificity, and reproducibility of DIA compared to MRM and PRM indicate that DIA is still more suitable for biomarker discovery instead of biomarker verification and validation, although DIA has advantages related to its analyte multiplexing capability and relative ease of assay development compared to MRM and PRM [122,123]. Therefore, the most widely used targeted methods are MRM and PRM, which can facilitate quantitation accuracy and reproducibility [124,125]. To achieve the desired accuracy and reproducibility, various parameters need to be optimized including precursor and product ion selection, collision energy and transmission settings for maximum sensitivity, and determination of liquid chromatography (LC) retention time [119,126]. In targeted quantitative proteomics methods, these are key concepts to obtain consistent peptide quantification metrics for proteins of interest in a large number of sample cohorts as well as inter-laboratory experiments.

MRM is a traditional targeted quantitative proteomics approach that is performed using triple quadrupole (QqQ) or quadrupole linear ion trap (QTRAP) instruments [127]. MRM uses the unique features of a QqQ or QTRAP instrument to detect the combinations of precursor and product ions that are termed MRM transitions (Q1/Q3 MRM ion pairs). The targeted specific precursor ions for peptides of interest are selected in the first quadrupole (Q1), and then transmitted into the collision cell (q2) for fragmentation. Finally, specific product ions from the targeted precursor ions are selected in the third quadrupole (Q3) for detection. Often, Q3 is a low-resolution mass analyzer, which cannot transmit ions with isolation widths <0.7–1.0 Da without losing sensitivity [119].

PRM is a newer targeted acquisition method that is performed with high resolution accurate mass (HRAM) mass analyzers such as Orbitraps (Thermo Scientific, Waltham, MA, USA). Compared to the QqQ-dependent MRM methods described above, in PRM methods, Q3 is substituted with an HRAM mass analyzer, which allows the parallel detection of all product ions from targeted precursor ions instead of selecting a limited number of product ions (typically three transitions) as is the case in MRM methods [128]. Therefore, PRM has several advantages compared to MRM. PRM provides highly specific spectra for all product ions from selected precursor ions, thereby permitting high confidence targeted peptide identification [128,129]. Since PRM uses several transitions to identify and quantify peptides, the predetermination of transitions and collision energy as required for MRM is not necessary for PRM, thus reducing the time required for method development. Compared to MRM, PRM provides significant improvement in signal-to-noise with high sensitivity. Interference from background ions is minimized when data are acquired using an HRAM mass analyzer [130]. These advantages of PRM suggest that PRM will be useful to characterize targeted peptides with post-translational modifications (PTMs) [125], which is difficult when using MRM. However, PRM has a relatively longer scan time for each targeted precursor ion compared to MRM, resulting in decreased efficiency when targeting multiple peptides simultaneously.

One of the most significant challenges in biomarker verification and validation using targeted quantitative proteomics approaches is the lack of sufficient sensitivity for detecting extremely low abundance proteins in body fluids. For example, the top 20 most abundant proteins in blood plasma/serum constitute approximately 99% of the total protein mass, while thousands of other proteins, including potential biomarker candidates, comprise 1% of the total protein mass and are present at concentrations as low as ng/mL or sub-ng/mL levels. Therefore, several methods have been developed to improve the detection capabilities in targeted quantitative proteomics approaches. These methods include immunoaffinity enrichment to enrich low abundance proteins or specific targeted proteins, immunoaffinity depletion to deplete high abundance interfering components, and fractionation to reduce the sample complexity [131]. These methods enable the improved detection of low abundance proteins by reducing the sample complexity.

Many regulatory agencies adopt the standards and guidelines published by the Clinical and Laboratory Standards Institute (CLSI) when setting regulatory guidelines for clinical laboratories. A forthcoming CLSI guidance document, “C64: Quantitative Measurement of Proteins and Peptides by Mass Spectrometry”, will provide a framework for developing clinical protein and peptide assays from conception to validation. To improve the translation of assays to clinical use, this guidance document focuses on the development of targeted mass spectrometry-based protein assays linked to clinically relevant analytical validation. The C64 guidance document provides broad recommendations for appropriately developing and validating quantitative protein and peptide assays for clinical applications while focusing on practical workflows and experimental strategies for developing and validating quantitative assays for soluble proteins and peptides in biofluids.

As mentioned in Section 3, “Overview of ovarian cancer biomarkers”, targeted quantitative proteomics approaches can bridge the chasm between candidate biomarker discovery and clinical utility. In biomedical research for personalized medicine, targeted quantitative proteomics methods can be used to develop biomarkers that are identified from untargeted global proteomics methods, and these targeted methods have potential use in screening individuals or patient cohorts according to disease risk or disease status [132]. This personalized approach can facilitate the early and highly sensitive detection of disease through long-term biomarker monitoring. In addition, targeted quantitative proteomics methods can be applied in systems biology research. Targeted quantitative proteomics methods can provide accurate quantitation of key proteins or protein complexes to provide insight into the dynamics of signaling pathways to understand the molecular mechanisms of signal transduction [133,134,135].

## 6. Future Perspectives

Targeted quantitative proteomics has become essential for the quantification of hundreds of biomarker candidates across several samples with high sensitivity, specificity, reproducibility, consistency, and accuracy [127,136,137,138]. Although antibody-based methods such as ELISA provide high sensitivity to quantify proteins, targeted quantitative proteomics can be used to overcome some of the limitations of these antibody-based methods including low specificity and selectivity. Targeted quantitative proteomics methods have been successfully used to verify and validate biomarker candidates in different types of cancer including ovarian [139,140,141,142], prostate [143,144], lung [145,146], colorectal [147], breast [148], liver [149,150,151,152], and pancreatic [153,154].

Despite great advances in targeted quantitative proteomics to facilitate the consistent identification and quantitation of peptides and proteins, the throughput of these methods is typically limited at ~50–100 proteins per single analysis. Compared to untargeted quantitative proteomics using DDA, targeted quantitative proteomics has a relatively low sample multiplexing ability. Achieving high-throughput measurements for hundreds of biomarker candidates will require advanced MS instrumentation that can provide throughput on the scale of DDA with the established advantages of targeted quantitative proteomics.

## 7. Conclusions

Untargeted and targeted quantitative proteomics have significantly improved biomarker discovery and validation efforts, respectively. Targeted quantitative proteomics approaches can bridge the gap between the discovery and validation phases in biomarker development in not only ovarian cancer, but also several other disease states for which there is an urgent need for biomarkers to enable early disease detection.

## Figures and Tables

**Table 1:** FDA-approved ovarian cancer biomarkers and biomarker candidates.

Biomarker *	Type	Phase(s)	Clinical Utility	Note ^§^
FDA-approved biomarkers				
CancerSEEK	Gene	4	Detection of genetic mutations	2019 FDA breakthrough device
CA125	Protein	4	Monitoring	Curated for phase 3 in breast
HE4	Protein	3	Early detection	
OVA1	Protein panel	3	Prediction	
Overa	Protein	5	Prediction	
ROMA	Protein panel	3	Prediction	
Biomarker candidates				
APC	Gene	3, 2		Under review in breast, lung, and prostate ^¥^
CDKN2A (p16)	Gene	2		Under review for phase 3 in breast and esophagus; under review for phase 1 in lung and prostate
EGFR	Gene	3		Curated for phase 3 in breast; under review for phase 3 in lung; under review for phase 1 in prostate
NID2	Gene	1		Curated for phase 1 in head and neck
p14/ARF	Gene	3, 2		Under review in prostate and ovary ^¥^
SMA4	Gene	3		
Cramer 5 marker panel	Protein panel	3		HE4, CA15-3, CA125, VTCN1, and CA72-4; Early detection
9 microsatellites	Protein	2		
ACKR3	Protein	2		
ACTR3	Protein	2		
ADAM12	Protein	2		
AFP	Protein	2		Certified by FDA in liver
AGRN	Protein	1		
AKT1	Protein	2		
AMBP	Protein	1		
AMY2A	Protein	3		
ANXA2	Protein	1		
APCS	Protein	3		
APOA1	Protein	3		Under review for phase 2 in breast and pancreas
APOB	Protein	3, 2		Under review in breast and ovary ^¥^
APOC4	Protein	3		
ARID1A	Protein	2		
ATP6AP2	Protein	1		
B2M	Protein	3		
BCAM	Protein	3		
BLVRB	Protein	3		
BRAF	Protein	2		
BRCA1	Protein	2		Under review for phase 2 in breast
BRCA2	Protein	2		
C3	Protein	1		
CA15-3	Protein	3		In ovarian cancer, used with CA125 for monitoring; Curated for phase 3 in breast; under review for phase 2 in lung; under review for phase 1 in prostate
CA19-9	Protein	3		In ovarian cancer, used with CA125 for monitoring; Curated for phase 3 in breast; under review for phase 3 in pancreas; under review for phase 1 in prostate
CA72-4	Protein	3		In ovarian cancer, used with CA125 for monitoring; Under review for phase 2 in breast
CADM1	Protein	3		
CBLC	Protein	3, 2		Under review in lung and ovary ^¥^
CCDC102B	Protein	2		
CCL11	Protein	3		Curated for phase 3 in breast
CD248	Protein	1		
CD59	Protein	1		
CDCP1	Protein	2		
CEACAM5	Protein	3		Curated for phase 3 in breast; under review for phase 2 in colon, lung, and pancreas; under review for phase 1 in prostate
CHI3L1	Protein	2		
CKM	Protein	1, 3		Under review in lung and ovary ^¥^
CPA4	Protein	1		
CRIP1	Protein	3		
CRIP2	Protein	2		
CRTAC1	Protein	1, 3		Under review in prostate and ovary ^¥^
CST6	Protein	1		
CTCFL	Protein	3		
CTGF	Protein	1		
CTNNB1	Protein	2		Under review in breast, pancreas, and ovary ^¥^
CXCL8	Protein	3		Curated for phase 2 in bladder; curated for phase 3 in breast; under review for phase 2 in lung; under review for phase 1 in prostate
DAG1	Protein	1		
DAPL1	Protein	3		
DEFB1	Protein	2		
DKK3	Protein	1		
DSC2	Protein	1, 2		Under review in prostate and ovary ^¥^
DSG2	Protein	1, 3		Under review in prostate and ovary ^¥^
ECM1	Protein	1		
EFEMP1	Protein	1		Under review for phase 3 in lung; under review for phase 1 for prostate
EFR3A	Protein	1		
EGFL6	Protein	2		
EMILIN2	Protein	1		
EPB41L3	Protein	2		
EPCAM	Protein	1		Target for cancer immunotherapy
EPSTI1	Protein	2		
ERBB2	Protein	3		Curated for phase 3 in breast; under review for phase 2 in colon and lung
ESM1	Protein	3		
FAM83H	Protein	2		
FAS	Protein	3		
FBLN1	Protein	1		
FBXW7	Protein	2		
FGFR2	Protein	2		
FGFR4	Protein	3		
FJX1	Protein	2		
FNDC3A	Protein	1		
FOLH1B	Protein	1		Under review for phase 1 in prostate
FOLR1	Protein	1		
FSH	Protein	3		
FSTL1	Protein	1		
FZD10	Protein	2		
GDF15	Protein	2		
GFPT1	Protein	3		
GH1	Protein	3		Under review for phase 2 in breast
GLOD4	Protein	1		
GM2A	Protein	1		
GPM6B	Protein	2		Under review for phase 1 in prostate
GPR158	Protein	3		
GPR39	Protein	1		
GPR65	Protein	2		
GRN	Protein	1		
H2AFJ	Protein	3		
H2AFV	Protein	3		
HAMP	Protein	3		
HAPLN1	Protein	1		Under review for phase 1 in lung
HIST1H2AA	Protein	3		
HMGB1	Protein	3		
HOXA9	Protein	2		Curated for phase 1 in head and neck; under review for phase 1 in prostate
HSPG2	Protein	1		
HTRA1	Protein	1		
ICAM1	Protein	2		Curated for phase 3 in breast; under review for phase 3 in prostate
IDH1	Protein	3		
IFI27	Protein	1		
IGF2	Protein	3		
IGFBP1	Protein	3		Under review for phase 2 in breast
IGFBP2	Protein	3		Under review for phase 2 in breast and colon
IGFBP3	Protein	1, 2		Under review in pancreas and ovary ^¥^
IGFBP4	Protein	1, 3		Under review in pancreas and ovary ^¥^
IGF-II	Protein	2		
IL10	Protein	3		
IL2RA	Protein	3		
IL6	Protein	2		Under review for phase 2 in breast
IL6R	Protein	3		
ITIH4	Protein	3		
KCP	Protein	3		
KLHL14	Protein	3		
KLK6	Protein	3		Used with CA125 for monitoring
KLK8	Protein	3		Under review for phase 2 in breast and lung
KRAS	Protein	1, 3, 2		Under review in colon, lung, pancreas, and ovary ^¥^
KRT19	Protein	3		Under review in prostate ^¥^
KRT8	Protein	1		
LAMA5	Protein	3		
LAMB2	Protein	3		
LAPTM4B	Protein	1		
LEP	Protein	3		Under review for phase 2 in breast
LGALS3BP	Protein	1		
LHB	Protein	3		
LPAR3	Protein	1		
LRG1	Protein	1, 2		Under review in breast, pancreas, and ovary ^¥^
LRRC47	Protein	3		
LTBP1	Protein	1		
LTBP2	Protein	1		
LY6G6C	Protein	3		
LZTS1	Protein	1		
MAPK1	Protein	2		
MIF	Protein	3		Under review for phase 1 in lung
MLH1	Protein	2		Curated for phase 3 in breast
MMP2	Protein	3		
MMP3	Protein	3		Curated for phase 3 in breast; under review for phase 1 in lung
MMP7	Protein	3		
MMP9	Protein	3		Curated for phase 2 in bladder; curated for phase 3 in breast; under review for phase in lung
MPO	Protein	3, 2		Under review in breast and ovary ^¥^
MPPED2	Protein	2		Under review for phase 1 in prostate
MPZL2	Protein	2		Under review for phase 1 in prostate
MSH2	Protein	2		
MSLN	Protein	3		
MXRA5	Protein	1		
NID1	Protein	3		
NMU	Protein	3		
NPC2	Protein	1		
NRAS	Protein	2		
NUCB1	Protein	1		
OLFML2B	Protein	1		
Osteopontin	Protein	3		Under review for phase 3 in breast; under review for phase 2 in liver; under review for phase 1 in lung
OVGP1	Protein	1		
P2RY14	Protein	2		
PCDH17	Protein	1		
PCOLCE	Protein	1		
PCSK9	Protein	3		
PEBP1	Protein	1		
PFAS	Protein	3		
PGGHG	Protein	3		
PI3	Protein	1		
PIK3CA	Protein	2		
PIK3R1	Protein	2		
PLEC	Protein	1		
PLTP	Protein	1		
PLXNB1	Protein	3		
PNP	Protein	3		
POLE	Protein	2		
POLQ	Protein	3		
POSTN	Protein	3		
PPBP	Protein	3		
PPP2R1A	Protein	2		
PRDX6	Protein	3		
PRL	Protein	3		Under review for phase 3 in breast
PRMT1	Protein	3		
PROS1	Protein	1		
PSAP	Protein	1		
PTEN	Protein	2		
PTH2R	Protein	3		
PTK7	Protein	3		
PTPRS	Protein	3		
QSOX1	Protein	1		
RMND5A	Protein	2		
RNF43	Protein	2		
SCGB2A1	Protein	2		Under review for phase 1 in prostate
SCNN1A	Protein	1, 3		Under review in prostate and ovary ^¥^
SDC1	Protein	2		Curated for phase 2 in bladder
SEC23B	Protein	2		
SECTM1	Protein	1		
SELENBP1	Protein	3		
SERPINA6	Protein	1		
SERPINE1	Protein	3		Curated for phase 2 in bladder; under review for phase 2 in breast
SLAMF8	Protein	2		
SLC11A1	Protein	1		
SLC30A6	Protein	2		
SLPI	Protein	3		Under review for phase 1 in lung and prostate
SMRP	Protein	2		Under review for phase 4 in lung
SOD3	Protein	3		
SPINT2	Protein	1		
SPON1	Protein	3		
SPON2	Protein	3		Under review for phase 2 in colon
SPP2	Protein	3		
ST13	Protein	3		
ST14	Protein	2		
TAGLN2	Protein	1		
TF	Protein	3		Under review for phase 1 in prostate
TNF	Protein	3, 2		Under review in breast, lung, and ovary ^¥^
TNFAIP1	Protein	2		
TNFAIP6	Protein	2		
TNFRSF1A	Protein	3		
TNFRSF1B	Protein	3		
TNFRSF21	Protein	3		
TNFRSF6B	Protein	3, 2		Under review in colon and ovary ^¥^
TP53	Protein	1, 2		Under review in breast, colon, lung, pancreas, prostate, and ovary
Transthyretin	Protein	3		
TSHB	Protein	3		
TSSK4	Protein	3		
VCAM1	Protein	3		Curated for phase 3 in breast
VCAN	Protein	3		
VTA1	Protein	2		
VTCN1	Protein	3		Used with CA125 for monitoring
VWF	Protein	1		
WNT10A	Protein	3		
WWC1	Protein	1		

* The information related to the biomarkers included in this table was obtained from the National Cancer Institute’s Early Detection Research Network (EDRN) website (https://edrn.nci.nih.gov/data-and-resources/biomarkers (accessed on April 28, 2021). ^§^ Additional information for each biomarker. ^¥^ No information for phase(s) according to organs.

## Data Availability

Not applicable.
